# Intensity modulated radiotherapy for locally advanced and metastatic pancreatic cancer: a mono-institutional retrospective analysis

**DOI:** 10.1186/s13014-014-0312-5

**Published:** 2015-01-10

**Authors:** Zheng Wang, Zhi-Gang Ren, Ning-Yi Ma, Jian-Dong Zhao, Zhen Zhang, Xue-Jun Ma, Jiang Long, Jin Xu, Guo-Liang Jiang

**Affiliations:** Department of Radiation Oncology, Fudan University Shanghai Cancer Center, 270 Dongan Road, Shanghai, 200032 China; Department of Pancreatic and Hepatobiliary Surgery, Fudan University Shanghai Cancer Center, 270 Dongan Road, Shanghai, 200032 China; Department of Oncology, Shanghai Medical College, Fudan University, 270 Dongan Road, Shanghai, 200032 China; Department of Radiation Oncology, Shanghai Proton and Heavy Ion Center, 4365 Kangxin Road, Shanghai, 201321 China

**Keywords:** Locally advanced pancreatic cancer, Metastatic pancreatic cancer, Concurrent chemoradiotherapy, Intensity modulated radiotherapy, Regional intra-arterial chemotherapy

## Abstract

**Background:**

To evaluate the role of intensity modulated radiotherapy (IMRT) for locally advanced pancreatic cancer (LAPC) and metastatic pancreatic cancer (MPC), and the prognostic factors in the setting of multidisciplinary approach strategies.

**Methods:**

63 patients with LAPC and MPC receiving IMRT in our institution were retrospectively identified. Information on patient baseline, treatment characteristics and overall survival (OS) time were collected. Data of pain relief and toxicity were evaluated. Univariate and multivariate analyses were conducted to investigate the prognostic factors.

**Results:**

All patients received IMRT with a median dose of 46.0 Gy. The median OS for LAPC and MPC patients were 15.7 months and 8.0 months, respectively (*p* = 0.029). Symptomatic improvements were observed in the 44 patients with abdominal/back pain after radiotherapy (RT) or concurrent chemoradiotherapy (CCRT), particularly in those with severe pain. Only 13.9% and 14.8% cases presented Grade ≥ 3 hematologic toxicities in RT and CCRT group, while no cases developed Grade ≥ 3 non-hematologic toxicities in both groups. Multivariate analysis indicated that tumors located in pancreas body/tail (HR 0.28, *p* = 0.008), pretreatment CA19-9 < 1000 U/mL (HR 0.36, *p* = 0.029) and concurrent chemotherapy (HR 0.37, *p* = 0.016) were independent favorable predictors for OS.

**Conclusions:**

CCRT further improved OS for LAPC and MPC with acceptable toxicities, and use of RT markedly alleviated pain. Tumors located in pancreas body/tail, pretreatment CA19-9 level of < 1000 U/mL and CCRT were associated with better OS. However, regional intra-arterial chemotherapy did not show any survival benefit in our study.

## Background

Pancreatic cancer is one of the deadliest human malignancies with a 5-year survival rate of less than 5% [1]. Although surgical resection offers the only opportunity for cure, only 20% of the patients are suitable for surgery at the time of diagnosis [2]. This dismal outcome is due to late stage diagnosis in the absence of specific early signs and symptoms, and also the high incidence of local and distant failures after treatments. Improving outcomes for pancreatic cancers continues to be a formidable challenge.

At the time of diagnosis, about 30% of patients present as locally advanced pancreatic cancer (LAPC), with a poor median survival of 10–12 months [3]. Currently, concurrent chemoradiotherapy (CCRT) is a conventional option for unresectable LAPC, but the role of irradiation remains controversial [4]. Historically, a randomized phase III study showed that CCRT offered survival benefit when compared with chemotherapy alone [5]. Nevertheless, other randomized trials did not confirm CCRT benefit [6,7], but those trials were criticized for the use of outdated radiotherapy (RT) technology, inappropriate total RT dose and the split-course RT, which had been abandoned. In a phase III randomized trial (ECOG-4201) to compare CCRT with chemotherapy alone in LAPC, which was closed early due to the poor accrual, for 74 patients enrolled in this study the median overall survival (OS) was significantly longer in the CCRT arm than that in chemotherapy alone [8]. Given that micrometastatic distant disease is most likely in LAPC patients, several months of induction chemotherapy have been proposed in order to exclude those, whose subclinical distant metastases become clinical during the induction chemotherapy and to pick up those appropriate patients, who would probably benefit from chemoradiotherapy. After several cycles of induction chemotherapy, CCRT will be delivered to the patients showing no evidence of disease progression. This approach could theoretically avoid unnecessary RT to the patients with subclinically distant metastases. However, relief of cancer-related symptoms and improving quality of life are also important for pancreatic cancers because of severe abdominal and back pain in a large percentage of patients. Therefore, RT would play an important role in pain relief for patients suffering from severe pain, even for metastatic pancreatic cancer (MPC).

The objective of this study is to evaluate the efficacy of RT and the prognostic factors in the setting of multidisciplinary approach strategies and to assess the effect of RT on pain relief in patients with LAPC and MPC.

## Methods

### Patients

This is a retrospective patient series and the patient selection criteria were: (1). Histologically or cytologically confirmed, or clinically diagnosed according to our clinical diagnosis criteria, which included typical pancreatic cancer symptoms (abdominal/back pain) and positive findings of CA19-9, CT, MRI and PET-CT; (2). Unresectable LAPC defined by the criteria of National Comprehensive Cancer Network (NCCN) guidelines (Version 1. 2013); (3). MPC suffering from abdominal and/or back pain caused by primary lesions and/or metastatic regional nodes; (4). Receiving RT alone or as a part of multidisciplinary approach; and (5). Medical record and follow-up data completed.

We found 69 consecutive patients matching the selection criteria from the medical record database in Fudan University Shanghai Cancer Center between May 2006 and April 2013. However, 63 patients were finally included in our analysis after excluding 6, who were lost to follow-up. Demographic and baseline characteristics, as well as treatment details for the entire group are summarized in Table 1. Of the 45 patients finally diagnosed by histology or cytology, 39 were adenocarcinomas, 5, poorly differentiated carcinomas and 1, acinar cell carcinoma. Initial symptoms of all 63 patients included abdominal/back pain (n = 41, 65.1%), weight loss (n = 13, 20.6%), epigastric discomfort (n = 8, 12.7%), jaundice (n = 3, 4.8%), anorexia (n = 2, 3.2%), pruritus (n = 1, 1.6%), diarrhea (n = 1, 1.6%), and fever/night sweat (n = 1, 1.6%). Seven LAPC patients (11.1%) with no specific symptoms were incidentally found, and finally confirmed by histology/cytology. Median diagnostic delay (interval from presenting symptoms to the diagnosis) was 2.0 months (range, 0.5 - 10.0 months). Pretreatment median levels of serum CA19-9 and CEA were 513.6 U/mL (range, 0.8 - 2111.0 U/mL) and 4.39 ng/mL (range, 0.5 - 149.9 ng/mL), respectively. For MPC patients, liver (17/32, 53.1%) was the most frequent metastatic site, and followed by lung (7/32, 21.9%), distant lymph node (4/32, 12.5%), peritoneal seeding (3/32, 9.4%), bone (2/32, 6.3%) and adrenal gland (2/32, 6.3%).

**Table 1:** **Characteristics of 63 pancreas cancer patients**

**Characteristics**	**No. of patients, n = 63**
Sex	
Male	43 (68.3%)
Female	20 (31.7%)
Age (Median)	62 (40–82)
Stage	
Locally advanced	31 (49.2%)
Metastatic	32 (50.8%)
Tumor location	
Head	23 (36.5%)
Body/tail	40 (63.5%)
Tumor size* (median)	4.6 cm (2.5 cm - 9.3 cm)
Diagnostic mode	
Histological/cytological	45 (71.4%)
Clinical	18 (28.6%)
CA19–9 at diagnosis	
≥ 1000 U/ml	25 (39.7%)
< 1000 U/ml	33 (52.4%)
Missing	5 (7.9%)
CEA at diagnosis	
>10 ng/ml	13 (20.6%)
≤10 ng/ml	41 (65.1%)
Missing	9 (14.3%)
Concurrent chemotherapy	
Yes	27 (42.9%)
No	36 (57.1%)
Systemic chemotherapy	
Yes	26 (41.3%)
No	37 (58.7%)
RIAC	
Yes	39 (61.9%)
No	24 (38.1%)

*Maximum diameter of the primary tumor.

*CA19-9* carbohydrate antigen 19–9, *CEA* carcinoembryonic antigen, *RIAC* regional intra-arterial chemotherapy.

### Treatment regimen

CCRT was strongly recommended for all patients without contraindication, and RT alone, as an alternative for those who refused CCRT. Among 63 patients 36 (57.1%) patients received RT alone, and 27 (42.9%), CCRT. Before and/or after RT, systemic chemotherapy, regional intra-arterial chemotherapy (RIAC) and the combination of the above were administrated in 26 (41.3%), 39 (61.9%) and 15 (23.8%) patients, respectively.

### Radiotherapy

All patients received intensity modulated radiation therapy (IMRT). The patients were immobilized by vacuum-lock with no breath control devices. For LAPC the gross tumor volume (GTV) encompassed the primary tumor and metastatic lymph nodes, which were shown on CT scan, MRI or PET-CT. For MPC only symptom-causing lesions including the primary tumor and regional metastatic lymph nodes were irradiated. The planning target volume (PTV) was defined as the GTV plus 1.0 - 1.5 cm in the craniocaudal direction and 0.8 - 1.0 cm in all other directions. The median total dose delivered to PTV was 46.0 Gy (range, 26.8 - 54.0 Gy) with a median fraction size of 1.8 Gy (range, 1.8 - 3.0 Gy). The conventional fractionation (1.8 - 2.0 Gy/fx) was used in 60 patients and 30 Gy in 10 fractions, in 3 MPC patients. The organs at risk (OAR) included stomach, duodenum, kidney, liver and spinal cord. Their RT dosimetric parameters are listed in Table 2.

**Table 2:** **Dosimetric parameters of radiation for the organs at risk**

**Organ at risk**	**Dosimetric parameter**	**Mean ± SD**	**Median**
Stomach	D_max_ (Gy)	51.29 ± 4.94	51.33
	V_50_ (%)	2.83 ± 4.40	0.46
Duodenum	D_max_ (Gy)	50.54 ± 4.92	51.26
	V_50_ (%)	5.44 ± 7.85	1.07
Right kidney	D_mean_ (Gy)	9.32 ± 3.74	10.40
Left kidney	D_mean_ (Gy)	10.74 ± 5.08	10.78
Liver	D_mean_ (Gy)	9.68 ± 4.80	8.81
Spinal cord	D_max_ (Gy)	31.57 ± 9.41	32.88

*D*_*max*_ maximum dose, *V*_*50*_ percentage of volume receiving more than 50 Gy, *D*_*mean*_ mean dose, *SD* standard deviation, *Gy* Gray.

### Chemotherapy

Among the 27 patients who received CCRT, the chemotherapy regimens were gemcitabine (GEM) (800-1000 mg/m^2^ on days 1, 8, 15, every 4 weeks) in 19 cases (70.4%), capecitabine (800 mg/m^2^, twice daily, Monday to Friday) in 4 (14.8%), and S1 (40 mg/m^2^, twice daily on days 1–14, every 3 weeks) in 4 (14.8%). For the 26 patients underwent neoadjuvant and/or adjuvant chemotherapy, the regimens included GEM 1000 mg/m^2^ on days 1, 8, 15, every 4 weeks (n = 16, 61.5%), GEM 1000 mg/m^2^ on days 1, 8 plus oxaliplatin 100 mg/m^2^ on day 1, every 3 weeks (n = 9, 34.6%) and S1 40 mg/m^2^, twice daily on days 1–14, every 3 weeks (n = 1, 3.8%). RIAC was given to 39 patients with a median cycle of 1 (range, 1–11). Briefly, 5-Fr Rosch hepatic catheter was inserted via the femoral artery using Seldinger’s technique, and the catheter position was verified by digital subtraction angiography. The chemotherapy regimen was 1000 mg/m^2^ of GEM and 500 mg/m^2^ of 5-FU. Two-thirds of the dosage was injected via celiac artery and one-third, the superior mesenteric artery.

### Follow-up and statistics

During the period of treatment patients were examined weekly, and after completion of treatment they were followed-up every 2 to 4 weeks for the first 3 months and every 3 months afterwards until the death. The toxicity was recorded using the Common Terminology Criteria for Adverse Events (CTCAE) version 3.0. The complete blood count and hepatic function was examined weekly during the treatment and on each follow-up visit after treatment completion. The pain was scored with the visual analogue scale (VAS), and was categorized into the none to mild (score 0–3), moderate (score 4–6) and severe pain (score 7–10). The serum CA19-9 and CEA was tested before and after treatment, and on each visit of the follow-up.

The pain relief and OS were the primary endpoints. OS was counted from the date of diagnosis and estimated by Kaplan-Meier method. Univariate analysis was conducted by the log-rank test. Variables trending towards significance (*p* < 0.10) on univariate analysis along with another plausible covariate, tumor size, were entered in multivariate analysis using Cox’s proportional hazard model. All of the statistical analyses were performed using SPSS Statistics version 20.0 (SPSS Inc., Chicago, IL, USA). *P* values less than 0.05 were considered statistically significant.

## Results

### Toxicity

Overall, the patients tolerated the treatments well. There were no treatment-related deaths. RT was terminated earlier than that planned in 3 patients (4.8%) when they had received 28.0 Gy, 30.0 Gy and 34.2 Gy, respectively due to the massive ascites resulting from tumor spreading in abdomen, refractory diarrhea or uncontrolled hyperglycemia. The observed RT-related and CCRT-related toxicities are listed in Table 3. All of non-hematological toxicity was scored as Grades ≤ 2 with the incidences of 44.4% (16/36) in RT and 59.3% (16/27) in CCRT. For overall hematological toxicity, rates of Grade 0, Grade 1–2 and Grade 3–4 events were 25% (9/36), 61.1% (22/36) and 13.9% (5/36) in RT group, and 11.1% (3/27), 74.1% (20/27) and 14.8% (4/27) in CCRT, respectively. In addition, CCRT had a statistically significant association with more neutropenia (*p* = 0.034) and thrombocytopenia (*p* = 0.019), and produced an increasing trend of elevated ALT (*p* = 0.051) compared to RT alone.

**Table 3:** **Comparison of toxicities between RT and CCRT groups**

**Type of toxicity**	**RT (n = 36), n**				**CCRT (n = 27), n**				***p*** **value** ^*^
	**Grade 1**	**Grade 2**	**Grade 3**	**Grade 4**	**Grade 1**	**Grade 2**	**Grade 3**	**Grade 4**	
Hematological									
Leukopenia	7	9	2	0	8	10	2	0	0.094
Neutropenia	2	3	1	0	9	2	0	1	0.034
Anemia	9	6	1	1	9	7	0	0	0.482
Thrombocytopenia	7	3	2	0	8	6	3	0	0.019
Non-hematological									
Hyperbilirubinemia	2	0	0	0	2	1	0	0	0.406
Elevated ALT	2	0	0	0	6	0	0	0	0.051
Elevated AST	2	0	0	0	2	1	0	0	0.406
Nausea/Vomiting	13	1	0	0	8	1	0	0	0.686
Diarrhea/Constipation	5	1	0	0	3	2	0	0	0.793

^*^Mann–Whitney U test, two-tailed.

*RT* radiotherapy, *CCRT* concurrent chemoradiotherapy, *ALT* alanine aminotransferase, *AST* aspartate aminotransferase.

### Pain relief

Forty-four patients (12 LAPC and 32 MPC) presented abdominal and/or back pain prior to RT, which included moderate pain in 32 patients and severe pain in 12. Pain relieved after RT or CCRT in all of the 44 patients, with no pain to mild pain in 40, and moderate pain in 4. The mean VAS score before RT or CCRT was 5.9 ± 1.1, and significantly declined to 2.1 ± 0.9 after RT or CCRT (*p* = 0.000). Patients who had severe pain showed a greater amelioration than those had moderate pain, with the decreased score of 5.0 ± 1.5 and 3.4 ± 0.8, respectively (*p* = 0.000).

### Overall survival

**Figure 1:**
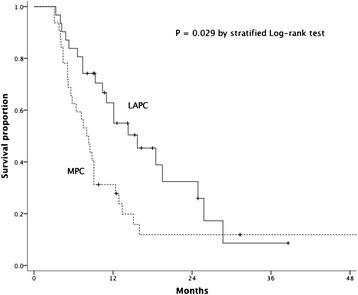
**Kaplan-Meier plot of overall survival for patients with locally advanced pancreatic cancer (LAPC) or metastatic pancreatic cancer (MPC).**

At the last follow-up visit in August 2013, 48 of 63 patients were dead and 15, alive. The median follow-up time was 9.1 months (range, 3.1 – 80.9 months) for all patients, and 14.2 months (range, 8.1 – 80.9 months) for the alive or the censored. The median OS time for all 63 patients was 9.3 months (95% CI, 6.4 – 12.2 months), and 15.7 months (95% CI, 9.6 – 21.8 months) and 8.0 months (95% CI, 6.2 – 9.8 months), for LAPC and MPC patients respectively (*p* = 0.029; Figure 1). The 1-year and 2-year OS rates for all patients were 46.3% and 19.4%, and 62.4% and 32.2% for LAPC, and 27.8% and 11.9% for MPC, respectively.

### Prognostic factors

**Figure 2:**
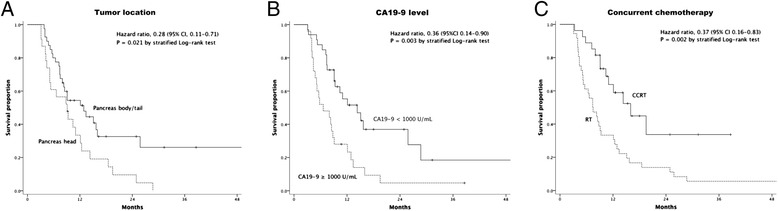
**Kaplan-Meier plots of overall survival stratified by independent prognostic factors. Panel A** shows pancreas head or body/tail cancer. **Panel B** shows pretreatment CA19-9 level ≥ or < 1000 U/mL. **Panel C** shows concurrent chemoradiotherapy (CCRT) or radiotherapy (RT) alone.

By univariate analysis, tumor located in body/tail, LAPC, pretreatment CA19-9 level of < 1000 U/mL, pretreatment CEA level of ≤ 10 ng/mL, systemic chemotherapy and concurrent chemotherapy were found to be significantly associated with longer OS (*p* < 0.05), whereas neither tumor sizes (*p* = 0.232), nor RIAC (*p* = 0.507) showed significant associations with OS (Table 4). Multivariate analysis indicated that tumors located in pancreas body/tail (HR 0.28, 95% CI 0.11 – 0.71, *p* = 0.008; Figure 2A), pretreatment CA19-9 < 1000 U/mL (HR 0.36, 95% CI 0.14 – 0.90, *p* = 0.029; Figure 2B) and concurrent chemotherapy (HR 0.37, 95% CI 0.16 – 0.83, *p* = 0.016; Figure 2C) were the independent favorable predictors for OS (Table 5).

**Table 4:** **Univariate analysis of factors affecting overall survival**

**Variable**	**Category**	**Univariate analysis**		
		**n**	**Median survival (months)**	***p*** **value** *
Gender	Male	43	9.1	0.109
	Female	20	14.3	
Age (years)	<65	36	9.1	0.623
	≥65	27	12.1	
Tumor location	Head	23	9.1	0.021
	Body/tail	40	12.9	
Tumor size (cm)	<4.5	22	12.1	0.232
	≥4.5	29	8.3	
	Missing	12		
Stage	Locally advanced	31	15.7	0.029
	Metastatic	32	8.0	
CA19-9 at diagnosis (U/mL)	≥1000	25	6.6	0.003
	<1000	33	14.3	
	Missing	5		
CEA at diagnosis (ng/mL)	>10	13	5.3	0.005
	≤10	41	9.3	
	Missing	9		
Systemic chemotherapy	Yes	26	18.5	0.001
	No	37	8.3	
Concurrent chemotherapy	Yes	27	16.0	0.002
	No	36	7.4	
RIAC	Yes	39	9.3	0.507
	No	24	10.4	

*Log-rank test, two-tailed.

*CA19-9* carbohydrate antigen 19–9, *CEA* carcinoembryonic antigen, *RIAC* regional intra-arterial chemotherapy.

**Table 5:** **Multivariate analysis of factors affecting overall survival**

**Variable**	**Category**	**Hazard ratio**	**95% CI**	***p*** **value** *
Tumor location	Body/tail	0.28	0.11 - 0.71	0.008
	Head	1		
CA19-9 at diagnosis (U/mL)	<1000	0.36	0.14 - 0.90	0.029
	≥1000	1		
Concurrent chemotherapy	Yes	0.37	0.16 - 0.83	0.016
	No	1		

*Multivariate analysis using the Cox proportional hazards model.

*CI* confidence interval, *CA19-9* carbohydrate antigen 19–9.

## Discussion

Pancreatic cancer incidence has increased dramatically in Shanghai, China. The age-standardized incidence rate has almost doubled in the past 3 decades with 3.38/100,000 in 1973 and 6.71/100,000 in 2009. The incidence of pancreas cancer ranked No. 7 in male and No. 8, in female, and the mortality was No. 4 for both genders. The patients suitable for surgery accounted for 10% to 15%, when diagnosed in China. In spite of the dismal outcome after surgery, it has been considered the sole modality for cure. For LAPC and MPC chemotherapy has been widely applied, but CCRT was also recommended as one option of the therapy. However, whether adding RT to chemotherapy could improve outcome remains questionable. Therefore we carried out this retrospective study to investigate the role of RT in LAPC and MPC.

In this study we selected the patients diagnosed by histology or cytology, but also those patients clinically diagnosed. The clinical diagnosis criteria included typical symptom of pancreatic cancer (abdominal and/or back pain) and all positive findings by CA19-9, CT and PET-CT, which yielded 100% of positive predictive value according to our recent study (unpublished data).

CCRT toxicity is the first one we have to consider. The data from a qualitative systematic review in 2009 demonstrated that CCRT increased OS when compared with exclusive RT, but was more toxic, in the management of LAPC [9]. Overall, the patients in our study tolerated therapy well in both groups. There were no statistically significant differences of toxicities between RT and CCRT, except for neutropenia and thrombocytopenia in our study.

As to the survival, a prospective randomized trial from Eastern Cooperative Oncology Group in 2011 reported that, for LAPC, the median OS, 1-year and 2-year OS rates were 11.1 months, 50% and 12% in patients treated with GEM-based chemoradiotherapy [10]. A more recent study retrospectively analyzed 109 cases of LAPC treated with 5-FU-based or GEM-based chemoradiotherapy, and their median OS was 12.1 months with 1-year and 2-year OS rates of 47% and 8%, respectively [11]. Our data revealed similar survival benefits (*p* = 0.002) with the median OS, 1-year and 2-year OS rates of 16.0 months, 58.6% and 33.5% for CCRT group, while 7.4 months, 30.6% and 11.1% for RT group. And by multivariate analysis, concurrent chemotherapy was confirmed as a beneficial factor for OS. These observations may further support the point of view that addition of concurrent chemotherapy to RT results in improved OS for patients with LAPC and MPC, with acceptable toxicities.

For LAPC the outcome in CCRT was better than that in RT, the median OS, 1-year and 2-year OS rates being 19.5 months, 71.1% and 40.6% versus 7.4 months, 35.7% and 14.3%, respectively (*p* = 0.019). However, for our 31 MPC patients addition of RT did not seem to have any contribution to improvement of OS. A recent prospective phase 3 trial conducted in 48 centers reported that the median OS and 1-year OS rate was respectively 6.8 months and 20.6% for MPC patients treated with single-agent GEM, whereas it increased to 11.1 months and 48.4% when FOLFIRINOX regimen was used [12].

On the other hand, for pancreas cancer, it is believed that relief of patients’ symptoms and abdominal and back pain is a main goal for palliation and improving patients’ quality of life. Overall, pain relief after RT or CCRT was significant. For the 44 patients experiencing pain prior to RT, pain VAS score reduced dramatically after therapy, especially for the patients with severe pain, who benefit most from therapy. Because of remarkable analgesic effect of RT, by which analgesic drug use could be decreased, the patients’ quality of life was improved. A previous study investigating the advantages and palliative effectiveness of hypofractionated RT in patients with locally advanced and metastatic adenocarcinoma of the pancreas also provided similar outcomes: pain improved in 70% of their cases and the median OS were 9 months for LAPC patients and 5 months for MPC patients [13].

In our study, a difference from the other studies in multidisciplinary approach was the use of RIAC in an attempt to increase locoregional control and to prevent liver metastasis based on a hypothesis, i.e., RIAC could probably give higher chemotherapy concentrations to the tumors and liver with a lower toxicity than systemic chemotherapy. Unfortunately, our data demonstrated that RIAC did not significantly improve outcome, compared with non-RIAC patients (9.3 months vs. 10.4 months). Our investigation of RIAC was inconsistent with that reported in the literature [14]. However, given the small sample of our study, the benefit from RIAC for pancreas cancer could not be definitively concluded.

For prognostic predictors of pancreas cancers, CA19-9 has been identified as the best tumor marker. Two prior retrospective studies demonstrated that higher level of pretreatment CA19-9 with the cutoff value of 420 U/mL and 400 U/mL, respectively, was unfavorable predictor for OS in LAPC patients who underwent CCRT [15,16]. Also, pretreatment levels of CA19-9 > 1000 U/mL was reported, in another study, to indicate a dismal survival for non-resectable patients [17]. Moreover, in a large prospective study of patients with advanced pancreatic cancer, including locally advanced and metastatic disease, CA19-9 level at diagnosis was shown to be an independent prognostic factor for OS and its cutoff value was defined as 59 times of the upper limit of the normal, which corresponded to about 2000 U/mL [18]. Thus, higher cutoff value of CA19-9 than its upper limit of the normal seems to be a reasonable watershed when we look for its prognostic significance, especially in advanced cases. Our study showed that pretreatment CA19-9 level was a significant prognostic factor by univariate analysis, the median OS being 14.3 months in patients with < 1000 U/mL, and 6.6 months, in ≥ 1000 U/mL (*p* = 0.003). Furthermore, as revealed by multivariate analysis, pretreatment CA19-9 ≥ 1000 U/mL was an independent indicator for poor OS.

In the literature, the location of pancreatic cancer could be generally divided into head and body/tail cancers with their respective clinical manifestations and maybe, different malignant potential [19,20]. There is increasing evidence that the anatomic location of pancreatic cancers seems to be a potential determinant of survival [21,22]. Interestingly, current data appear to draw opposite conclusions. Data from SEER in USA including 24,648 cases revealed that patients with pancreatic head cancer had a statistically improved median OS, compared to those with pancreatic body/tail cancer (6 months versus 4 months, *p* < 0.001) [23]. In contrast, data from Eastern country, the National Pancreatic Cancer Registry of Japan, including 7,417 cases presented that 5-year OS for patients with pancreas head cancer was significantly lower than that for those with pancreas body/tail tumor (10.7% versus 13.8%, *p* = 0.001) [24]. Our data revealed similar results with that of Japan. The median OS in pancreatic head cancer group and body/tail cancer group were 9.1 months and 12.9 months, respectively (*p* = 0.021). And tumors located in pancreas head was finally identified as an independent risk factor for poor prognosis by multivariate model.

## Conclusions

In conclusion, CCRT yielded a better OS than RT in LAPC and MPC patients, and moreover, was well tolerable. Pain relief was significant, particularly in patients with severe abdominal and back pain. Adding RIAC to RT or CCRT did not show any survival benefit. The favorable prognostic predictors were pancreas body/tail cancer, CA19-9 level of < 1000 U/mL at diagnosis and CCRT. Our study indicated that CCRT could be recommended to LAPC, and also MPC patients suffering from abdominal and/or back pain. RT is also a treatment option for those who could not tolerate, or refuse CCRT.
